# Estimation of Relative Risk of Mortality and Economic Burden Attributable to High Temperature in Wuhan, China

**DOI:** 10.3389/fpubh.2022.839204

**Published:** 2022-02-16

**Authors:** Si Chen, Junrui Zhao, Soo-Beom Lee, Seong Wook Kim

**Affiliations:** ^1^School of Resources and Environmental Science, Hubei University, Wuhan, China; ^2^Department of Transportation Engineering, University of Seoul, Seoul, South Korea; ^3^Department of Applied Mathematics, Hanyang University, Ansan, South Korea

**Keywords:** high temperature, mortality relative risk, economic burden, meta-analysis, value of statistical life (VSL)

## Abstract

In the context of climate change, most of the global regions are facing the threat of high temperature. Influenced by tropical cyclones in the western North Pacific Ocean, high temperatures are more likely to occur in central China, and the economic losses caused by heat are in urgent need of quantification to form the basis for health decisions. In order to study the economic burden of high temperature on the health of Wuhan residents between 2013 and 2019, we employed meta-analysis and the value of statistical life (VSL) approach to calculate the relative risk of high temperature health endpoints, the number of premature deaths, and the corresponding economic losses in Wuhan City, China. The results suggested that the pooled estimates of relative risk of death from high temperature health endpoints was 1.26 [95% confidence interval (CI): 1.15, 1.39]. The average number of premature deaths caused by high temperature was estimated to be 77,369 (95% CI: 48,906–105,198) during 2013–2019, and the induced economic losses were 156.1 billion RMB (95% CI: 92.28–211.40 billion RMB), accounting for 1.81% (95% CI: 1.14–2.45%) of Wuhan's annual GDP in the seven-year period. It can be seen that high temperature drives an increase in the premature deaths, and the influence of high temperature on human health results in an economic burden on the health system and population in Wuhan City. It is necessary for the decision-makers to take measures to reduce the risk of premature death and the proportion of economic loss of residents under the impacts of climate change.

## Introduction

Climate change poses a threat to human health, which through direct effects including increased frequency of high temperatures, floods, droughts and severe storms, and indirectly through impacts on ecosystems (1). The first part of Sixth Assessment Report of the Intergovernmental Panel on Climate Change (IPCC) finished by Working Group I has reported that the current global average surface temperature is about 1°C above pre-industrial levels, and the global surface temperature will continue to rise until at least the middle of this century. If greenhouse gas emissions are not reduced in the future, global temperature will be likely to exceed 1.5 and 2°C by the end of twenty-first century (2). As the global temperature rises, the intensity and frequency of extreme heat events are rapidly increasing.

Most regions are already suffering from extreme heat, which has had significant impacts on human health. Some epidemiological studies have illustrated that exposure to high temperatures results in cardiovascular disease, respiratory disease and cerebrovascular disease (3). Extreme heat not only leads to a range of diseases but also results in mortality. In California during 2006 and Wisconsin from July 16 to July 18 in 2012, there were 655 premature deaths and 27 deaths, respectively (4, 5) due to heat. A report published by The Oregonlive on July 1, 2021, announced that at least 63 people had died in Oregon from the heat, and Multnomah County, where the largest number of deaths occurred, had received 491 emergency medical calls in 1 day (6). Moreover, the death toll in Oregon had risen to 107 in the next week (7). Global News also reported on July 29, 2021 that 570 people died from heat-related deaths in British Columbia (8). From 2000 to 2019, Asia, Africa, and the global excess deaths due to high temperatures were 224,022, 25,549 and 489,075, respectively (9). Exposure to high temperature in addition to the health risks, which can lead to premature death, also places a burden on the economy. Several economically developed countries have quantified the economic costs of health hazards from high temperatures, and according to data regarding Michigan from 1971 to 2000 and California in 2006, the economic losses due to heat-related deaths were about $4.2 million and $5.1 billion, respectively (4, 10). Moreover, a study on heat-related deaths in Zaragoza, Spain showed that the hospitalization costs for these deaths amounted to €426,087 in 2002–2006 (11). From 2013 to 2014, Australia experienced a further economic burden of $6.2 billion per year due to reduced labor productivity as a result of heat stress (12).

Heat-related deaths have also increased along with global temperatures. Among the five continents in addition to Antarctica, Asia, specifically Southern and Eastern Asia ranked first and third in the number of excess deaths due to high temperature, respectively (9). Because of their higher vulnerability and limited capacity to adapt to high temperatures, developing countries are more likely to be exposed to heat-related health threats than developed countries (13) and therefore are desperately in need of a quantitative health outcomes analysis. In contrast, China, one of the developing countries, is very limited on articles regarding quantitative analysis of high-temperature health outcomes. The frequent activities of tropical cyclones formed over the western North Pacific Ocean affect the climate in East and Southeast Asia, while indirectly leading to an increase in the number of high-temperature days in the middle and lower reaches of the Yangtze River region in the east-central region (14). In the 60 years from 1955 to 2014, central China was the most severely threatened by heatwaves (a type of hot weather) and had the highest frequency and number of annual heatwaves (15). Information on the economic burden of heat is also in urgent need in the central region, which is most affected by high temperature. Therefore, it is most fitting to consider Wuhan, the largest city in central China, in a case study to assess the economic losses of premature death caused by high temperature in 2013–2019.

The purpose of this paper is to: (1) calculate the overall estimate value by pooling the relative risk (RR) effect values of heat-related disease deaths from previous studies in Wuhan; (2) assess the burden of heat-related disease deaths on the GDP of Wuhan using the value of statistical life (VSL) method; (3) propose management countermeasures to assist policymakers in establishing a heat warning system that reduces heat exposure mortality among the population, especially vulnerable populations, and improving the quality and level of public health management.

## Methods

### Study Area

**Figure 1:**
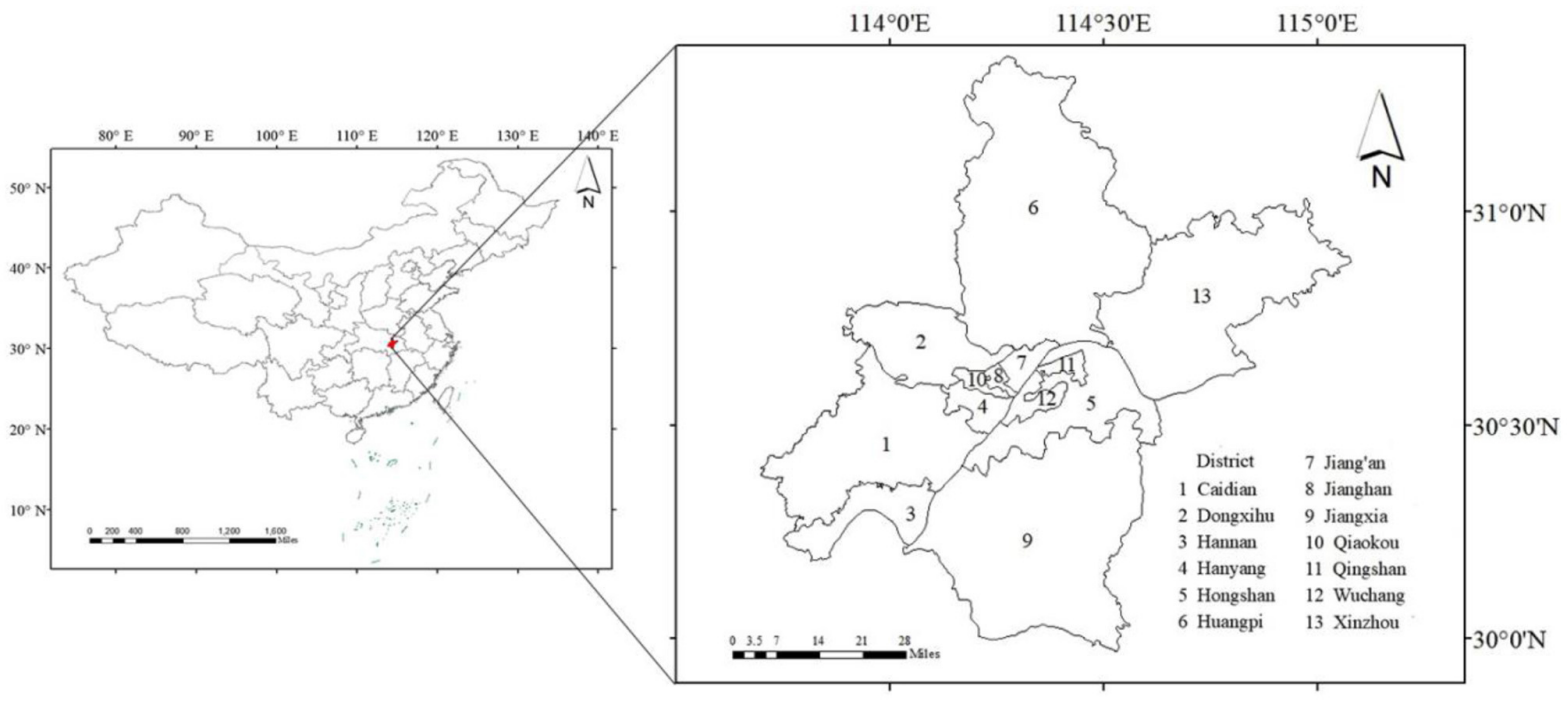
Geographical location of Wuhan.

Wuhan City is the capital of Hubei Province (29°58′N to 31°22′N,113°41′E to 115°05′E) and a total area of 8,494 km^2^. Located in the subtropical high-pressure belt, Wuhan has the famous title of a “furnace” city. From 1951 to 2018, there were 386 days when the daily maximum temperature in Wuhan exceeded 35°C, and the days with a maximum temperature of 38°C accounted for about 2.8% of the warm season (May to September). As the largest city in central China, Wuhan ranked fourth and third among 15 sub-provincial cities in China in terms of total GDP in 2013 and 2015, respectively. Its GDP topped central China in 2014, and it was selected as a new first-tier city in 2019. The study of heat-related diseases and deaths, as well as their economic burden in Wuhan, can alert policymakers to mitigate the health and economic losses caused by high temperatures, which is significant for the city to accelerate its development as a national economic center. The geographical location of Wuhan within China and the map of the city are shown in Figure 1.

### Overall Study Description

**Figure 2:**
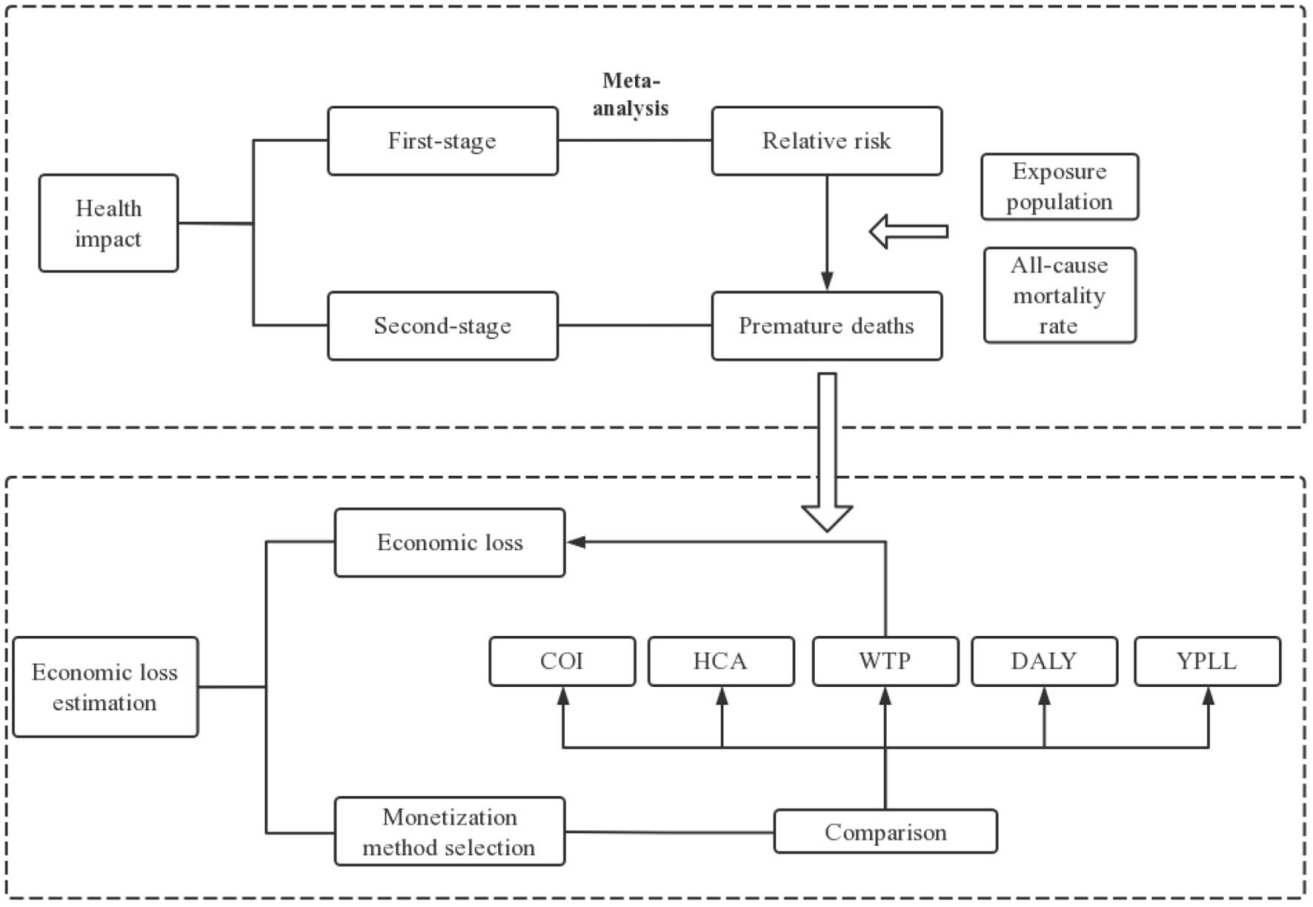
Research route flowchart.

High-temperature weather is a health threat and leads to premature death in the population. In this paper, with the context of health risk assessment being widely applied to provide a quantitative evaluation of the potential negative impact of hazards on human health (16), a two-stage analysis combined with meta-analysis were applied to calculate the relative risk of exposure to high temperature and the number of premature deaths successively. Further discussed are five monetization methods to estimate the economic losses caused by premature death, with the most optimal one selected and combined with health impacts to derive health economic losses. The research flowchart is shown in Figure 2.

### Estimation of Health Impacts

In this part, a two-stage analysis was used to estimate the health effects and premature deaths from exposure to high temperatures. In the first stage, due to unavailability of epidemiological data, we extracted and pooled the estimates of relative risks (RRs) from searched and screened literature. A study of collecting heavy metal and metalloid from surface soils in central China during 2007–2017 also collected data from various literature for the same reason (17). The number of premature heat deaths from the overall RRs was calculated in the second stage.

#### First-Stage Analysis

Since most current studies on heat-related deaths have focused on cardiovascular disease, cerebrovascular disease, respiratory disease, ischemic heart disease, and non-accidental death (3), we used these five major causes of heat-related death in the first stage as health endpoints.

Then the literature was searched on CNKI, PubMed, Web of science and Wanfang Data. The search key words combined “China/Chinese/Hubei/Wuhan”, “High temperature/Heat/Heat wave/Hot temperature/Extreme temperature” with “death/mortality” and “relative risk/RR (relative risk)/increase risk.” To ensure a more complete coverage of the literature, we also consider the references of the searched literature. The searched literature were screened according to the following inclusion and exclusion criteria.

Inclusion criteria are (1) studies on the relationship between high temperature and death in five health endpoints and (2) effect estimates and corresponding 95% confidence interval for the risk of death from the high temperature on health endpoints such as Odds Ratio (OR), Relative Risk, and percent change which can be converted into RR.

Exclusion criteria are (1) overview literature pieces, (2) No Wuhan City as a study area, and (3) duplicate literature.

The final literature was determined by viewing the titles and abstracts of the literature as well as the entire literature from start to finish. Then data from the included literature was extracted to make a characteristics summary table. The extracted data include title, author, study period, type of death, and effect estimates. Moreover, when the effect estimate is shown as percentage change, it is converted to RR using Equation (1), or, if it is shown as OR, using Equation (2):


(1) $$\begin{array}{r} RR=\frac{percent\;change}{100}+1 \end{array}$$

(2) $$\begin{array}{r} RR=\frac{OR}{\left(1-P_{0}\right)+\left(P_{0}\times OR\right)} \end{array}$$

where RR is relative risk, *OR* is Odds Ratio, *P*_0_ is the incidence of disease in non-exposed populations.

Finally, we calculated the heterogeneity (I^2^) and pooled RRs with either a random effect model if I^2^ > 50%, or a fixed effect model if otherwise. When pooling RR, we chose the largest estimates of RR (18). The process of pooling RRs was performed using Stata SE version 15.

#### Second-Stage Analysis

With the above RR estimates, Equation (3) was used to assess the number of premature deaths due to high temperature (19).


(3) $$\begin{array}{l} \begin{array}{ccc} P & = & y_{0}\times Pop\times (\frac{RR-1}{RR}) \end{array}\\ \;\begin{array}{cc} = & y_{0}\times Pop\times AF \end{array} \end{array}$$

where P (person) is premature deaths, *y*_0_ (‰) is the baseline rate of all-cause death, Pop (person) is the exposed population, RR is the relative risk of health endpoints mortality due to high temperature, and AF is the attributable fraction of high temperature to death. The values of *y*_0_ (‰) and Pop (person) were obtained from Wuhan statistical yearbook (20) (details in Table 1), and the RR is derived from the overall RR estimated by the first-stage analysis.

**Table 1:** Exposed population and all-cause mortality rate in Wuhan between 2013 and 2019.

**Year**	**2013**	**2014**	**2015**	**2016**	**2017**	**2018**	**2019**
Exposed population (person)	8,220,493	8,273,117	8,292,666	8,338,450	8,536,517	8,837,299	9,063,973
All-cause death rate (‰)	4.98	4.97	5.75	5.44	11.62	5.52	5.72

### Economic Loss Estimation

Current studies on the monetization of health hazards caused by environmental issues consist of the cost of illness (COI) approach (21), the human capital approach (HCA) (22), the modified human capital approach [Years of Potential Life Lost (YPLL)] (23), disable adjusted life years (DALY) (22), and the willingness to pay approach (WTP) [i.e., value of statistical life (VSL)] (21). COI is a calculation of the costs incurred by the disease; however, it is deficient in assessing health loss from premature death. First, it targets people as research subjects and has a large amount of data, which can easily lead to incomplete data during the survey and thus affect the accuracy of the final results. Second, it considers only all costs caused by diseases without taking into account the health preferences of affected individuals (23). HCA refers to capital embodied in workers, while non-labor force populations such as the out-of-work and elderly populations are considered less valuable because they have no income at the time of death. There are ethical and moral flaws in this approach (23). The modified human capital approach considers per capita GDP as a statistical life year contribution to society, which differs from the human capital approach in that it considers the contribution of the labor force to the socio-economy from the perspective of society as a whole, but it may have a significant regional variation due to the difference of per capita GDP (24). DALY measures the difference in quality of life between a disabling health condition and a normal health condition. This method lost uniformity in the choice of weights corresponding to different levels of incapacity when the weighting method of health-related quality differences was introduced into the calculation, and it has limitations in age and gender weights (25). WTP is the amount people are willing to pay to reduce a certain level of health hazard, and VSL is the quotient of the amount paid and the risk of hazard reduction. Willingness-to-pay based on the contingent valuation method (CVM) allows flexible assessment of environmental health losses of the population (26).

Based on the characteristics of the above methods, this study adopted the willingness-to-pay (i.e., value of statistical life) method to calculate the economic loss of the health endpoints of heat-related death. Due to the lack of VSL value related to high temperature, Adélaïde et al. analyzed health-related economic impacts in 96 French metropolitan areas during heat waves from 2015 to 2019 and proposed that some values such as VSL in the context of air pollution could be relied upon health-related economic losses estimation (27). Although the VSL of air pollution is available from various research, there is little information of a ready estimate for Wuhan City found from published works up to now. In this paper, a total of three different paths to obtain VSL estimate are proposed as follows:

(1) Employing meta-analysis to obtain VSL;
(2) Based on the WTP method calculated according to the following formula:

(4) $$\begin{array}{r} VSL=\frac{WTP}{\Delta P} \end{array}$$

where WTP is the amount of money residents are willing to pay to reduce the risk of death; ΔP is a certain risk of death reduction;

(3) Converting VSL data of known cities into VSL of study cities by benefit transfer (BT) approach (28):

(5) $$\begin{array}{r} VSL_{WH,k}=VSL_{city,i}\times {\left(\frac{I_{WH,k}}{I_{city,i}}\right)}^{\beta } \end{array}$$

where VSL_WH, k_ (million RMB/person) is the VSL of Wuhan in year *k*; VSL_city, i_ (million RMB/person) is the VSL of city in year *i*; *I*_WH, k_ (RMB/person) and *I*_city, i_ (RMB/person) are the per capita disposable income of Wuhan and city in year *k* and *i* respectively; and β is the income elasticity of VSL (the income elasticity of 0.8 was recommended by the Organization for Economic Co-operation and Development (29).

Once the VSL estimate could be obtained from the selected method, the economic value of environmental hazards, such as air pollution, to mortality mitigation is the product of estimates of “statistical lives saved” and “statistical value per life” (30). Thus, the economic value of high-temperature loss is the product of the “number of premature deaths” and “statistical value per life.” The health economic loss can then be calculated using Equation (6) (19):


(6) $$\begin{array}{r} EC_{al}=P\times VSL \end{array}$$

where *P* (person) is premature deaths; VSL (million RMB/person) is statistical value per life, and EC_al_ is high temperature all-cause premature death economic loss.

## Results

### Literature Selection and Summary

**Figure 3:**
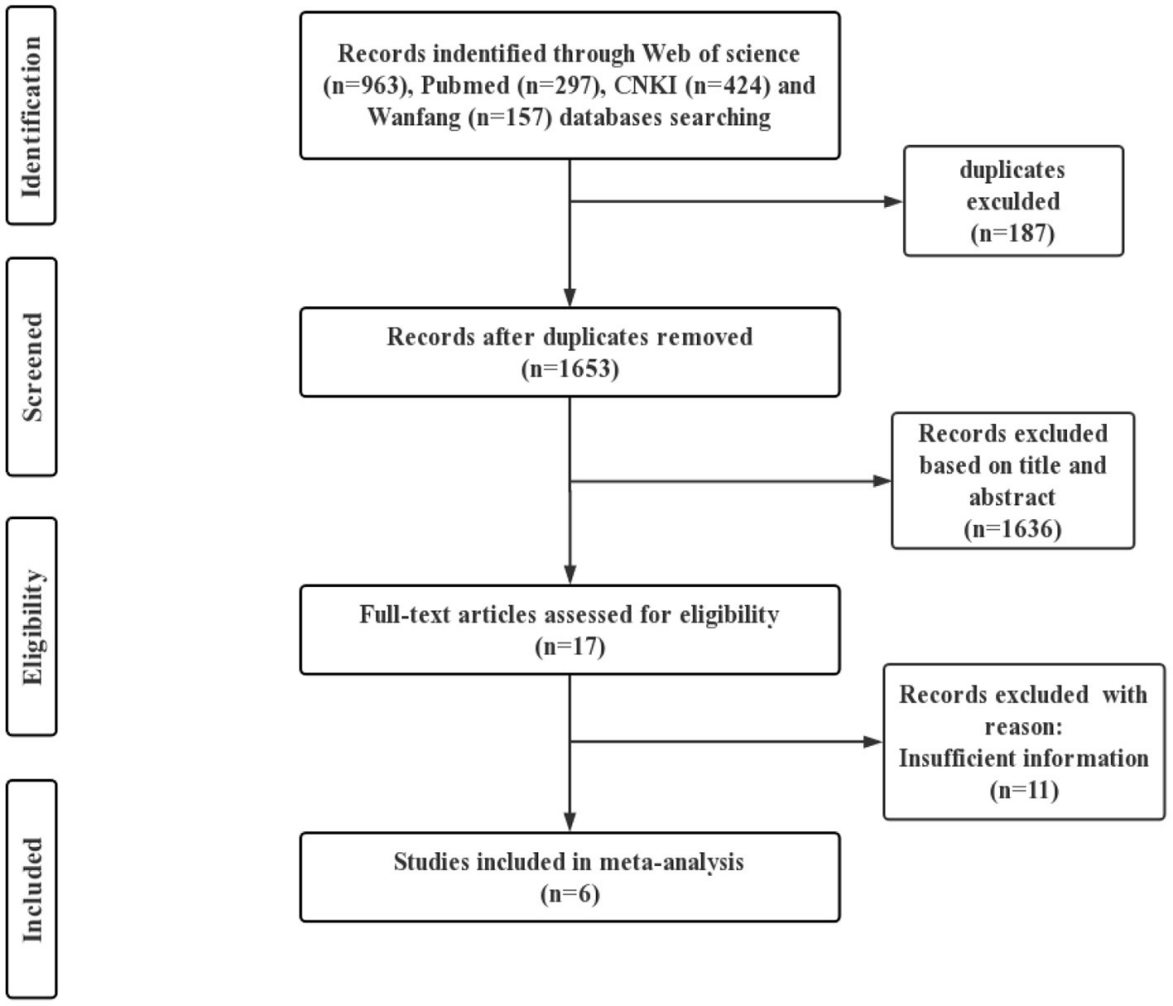
Flow chart of literature selection.

A total of 1,841 publications were identified initially from Web of science (963), Pubmed (297), CNKI (424) and Wanfang (157) databases search. After removing duplicate publications, a total of 1,653 articles have remained. Of those, 17 publications fit the exclusion and inclusion criteria based on the study titles and abstracts. Among them, 6 papers were determined to have sufficient information and therefore finally included in meta-analysis, with 5 papers in English and 1 in Chinese (Figure 3). The summary of the included literature on the risk of death from high temperature on health endpoints in Wuhan are listed in Table 2.

**Table 2:** Summary of the characteristics of included studies.

**References**	**Study period**	**Mortality event**	**RR (95% CI)**
Ma (31)	2003–2005	Cardiovascular disease	1.14 (1.04,1.24)
Ma (31)	2003–2005	Respiratory disease	1.11 (0.88,1.39)
Guo et al. (32)	2004–2008	Ischemic heart disease	1.59 (1.16,2.18)
Zhang et al. (33)	2003–2010	Cardiovascular disease	1.34 (1.26,1.43)
Zhang et al. (33)	2003–2010	Respiratory disease	1.57 (1.24,1.98)
Zhang et al. (33)	2003–2010	Cerebrovascular disease	1.69 (1.41,1.95)
Zhang et al. (33)	2003–2010	Ischemic heart disease	1.17 (1.08,1.27)
Zhang et al. (33)	2003–2010	Non-accidental	1.25 (1.19,1.32)
Wu et al. (34)	2003–2010	Ischemic heart disease	1.15 (1.01,1.30)
Bao et al. (35)	2008–2011	Non-accidental	1.35 (1.18,1.55)
Zhang et al. (36)	2004–2008	Cerebrovascular disease	1.01 (0.98,1.05)

### Relative Risk of High Temperature Exposure and Premature Deaths

**Figure 4:**
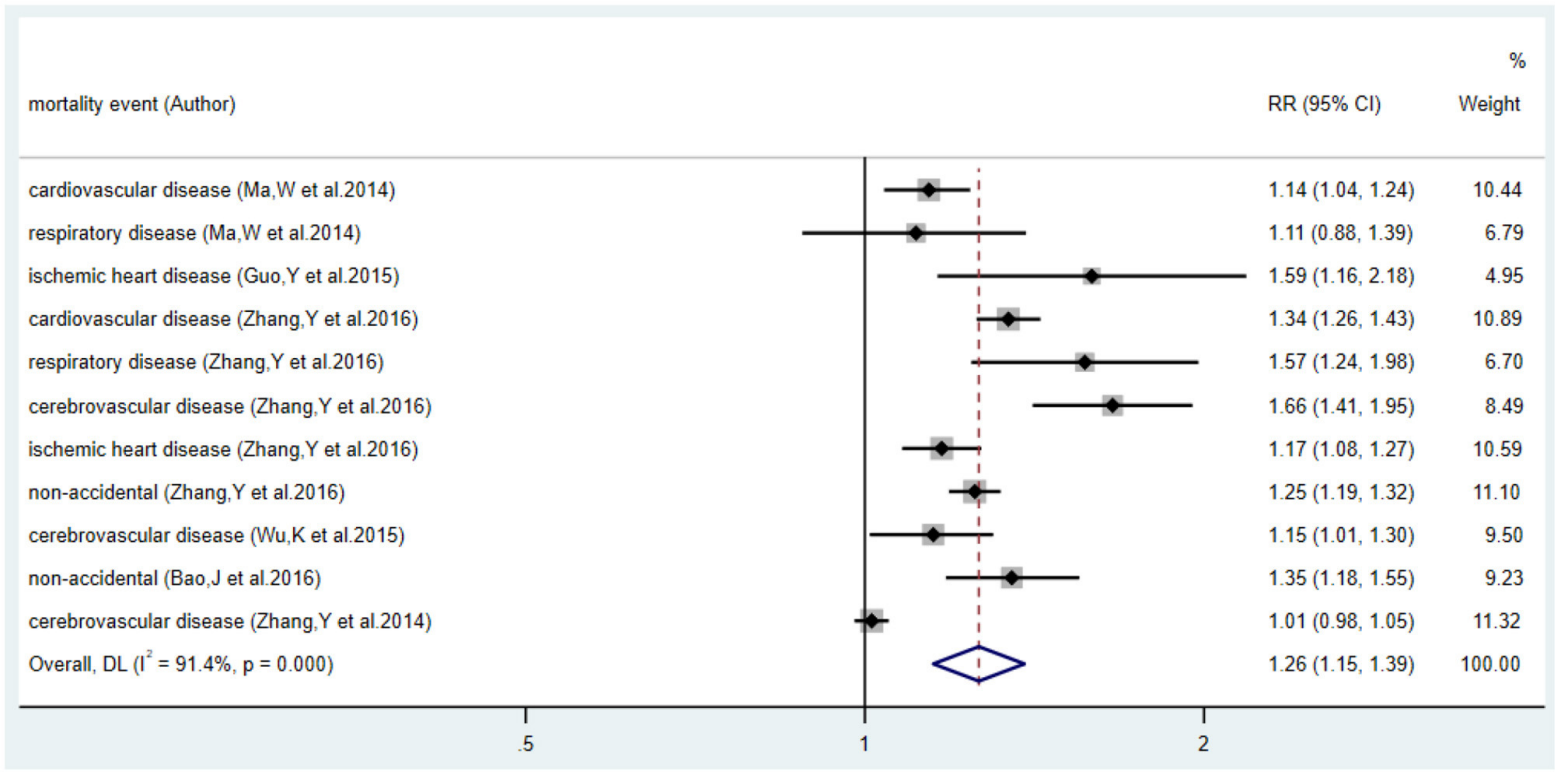
Forest plot of relative risk and 95% CI for relationship between the high temperature and health endpoints.

The estimates of RR of each study and overall pooled RR are shown in Figure 4, and the results showed statistical significance (*p* < 0.05). Since the significant heterogeneity (*I*^2^ = 91.4 %) was detected, we chose the random effect model to pool the RR. The pooled estimate of RR (95% CI) was 1.26 (95% CI: 1.15, 1.39), which suggests that the risk of mortality from residents' health endpoints will increase by 26% when exposed to high temperatures.

The number of premature deaths due to high temperatures in Wuhan based on the calculation of Equation (3) is presented in Table 3. From 2013 to 2019, this number had noticeable fluctuations, rising as high as 77,369. It trended downward from 2013 to 2014 and from 2015 to 2016 but rose sharply after 2016. It peaked in 2017 and fell again in 2018, yet it remained larger than that in 2013.

**Table 3:** Premature deaths (95%CI) due to high temperature in Wuhan, 2013–2019.

**Year**	**Premature death (person)**	**Year**	**Premature death (person)**
2013	8,848 (5,340–11,487)	2017	20,469 (12,939–27,832)
2014	8,485 (5,364–11,537)	2018	10,067 (6,363–13,688)
2015	9,840 (6,220–13,379)	2019	10,699 (6,763–14,547)
2016	9,361 (5,917–12,728)	2013–2019	77,369 (48,906–105,198)

It should be noted that the number of exposed populations as well as mortality rates in Wuhan varied from 2013 to 2019, and the number of premature deaths determined by these two variables also differed annually. The abnormal number of premature deaths in 2017 was attributed to an abnormal all-cause mortality rate for that year, which was more than twice as high as the rate for the other years over the seven years. In the same year, the mortality rate in Changsha City was similarly abnormal. According to the 2017 Changsha City National Economic and Social Development Statistical Bulletin, the Municipal Public Security Bureau verified the information of people who died but did not cancel their household registration and cumulatively canceled the household registration of these people, causing the mortality rate that year to be higher than usual (37). In this regard, we reasonably assumed that Wuhan City also carried out the same cancellation action in 2017, resulting in an abnormal mortality rate and further causing a high number of premature deaths from high temperature as well.

### Value of Statistical Life Obtainment

In this study, three methods were considered to estimate VSL: meta-analysis, willingness-to pay (WTP) and benefit transfer (BT) methods. As for the meta-analysis method, with the wide application of meta-analysis in the field of environmental economics, Xu et al. (38) assessed four publications relevant to the air pollution CVM with meta-regression model to estimate the value of air pollution statistics life in China to be about 86 million RMB. More evidence suggests that income level is positively associated with VSL and is the main factor influencing VSL (39, 40). The time series study in this paper is comprised of time variation of VSL with per capita income. Thus, fixed meta-analysis value cannot be generalized for use in a time series analysis.

It is feasible to obtain VSL based on Equation (4) by using the WTP method. Gao et al. surveyed the willingness to pay for air pollution health risk reduction in three of the six main urban areas of Beijing measured by contingent valuation method, with VSL values ranging from 0.667 to 1.1 million RMB (41). Similarly, Xu estimated the VSL value for Hangzhou residents in 2004 to be 2.218 million RMB (40). A More recent study by Peng et al. estimated the value of statistical life in Chongqing and Sichuan to be 3.928 million and 4.02 million RMB, respectively, with the single-boundary dichotomous function model of the CVM (42). Unfortunately, we failed to find any willingness-to-pay surveys on air pollution mortality risk reduction carried out in Wuhan city.

The BT method is meant to convert from a specific VSL study that has been estimated for a particular city, based on an exponential linear relationship of the proportion of income levels per capita. Compared to the previous methods, the BT method reduces the consumption of human, material, and financial resources (40) as well as takes the temporary differences in income earnings levels into account, therefore considered the most appropriate method for this study.

When selecting the VSL values of domestic and foreign regions, some scholars discovered that the value of domestic VSL was generally lower than latter (38, 39). We speculate that it is caused by the difference in economic development in China and foreign countries. Therefore, in this paper, VSL of domestic cities was selected, specifically the VSL of Beijing in 2010 (1.68 million RMB) based on Xie (43).

### Health Economic Loss Attributed to High Temperature

**Figure 5:**
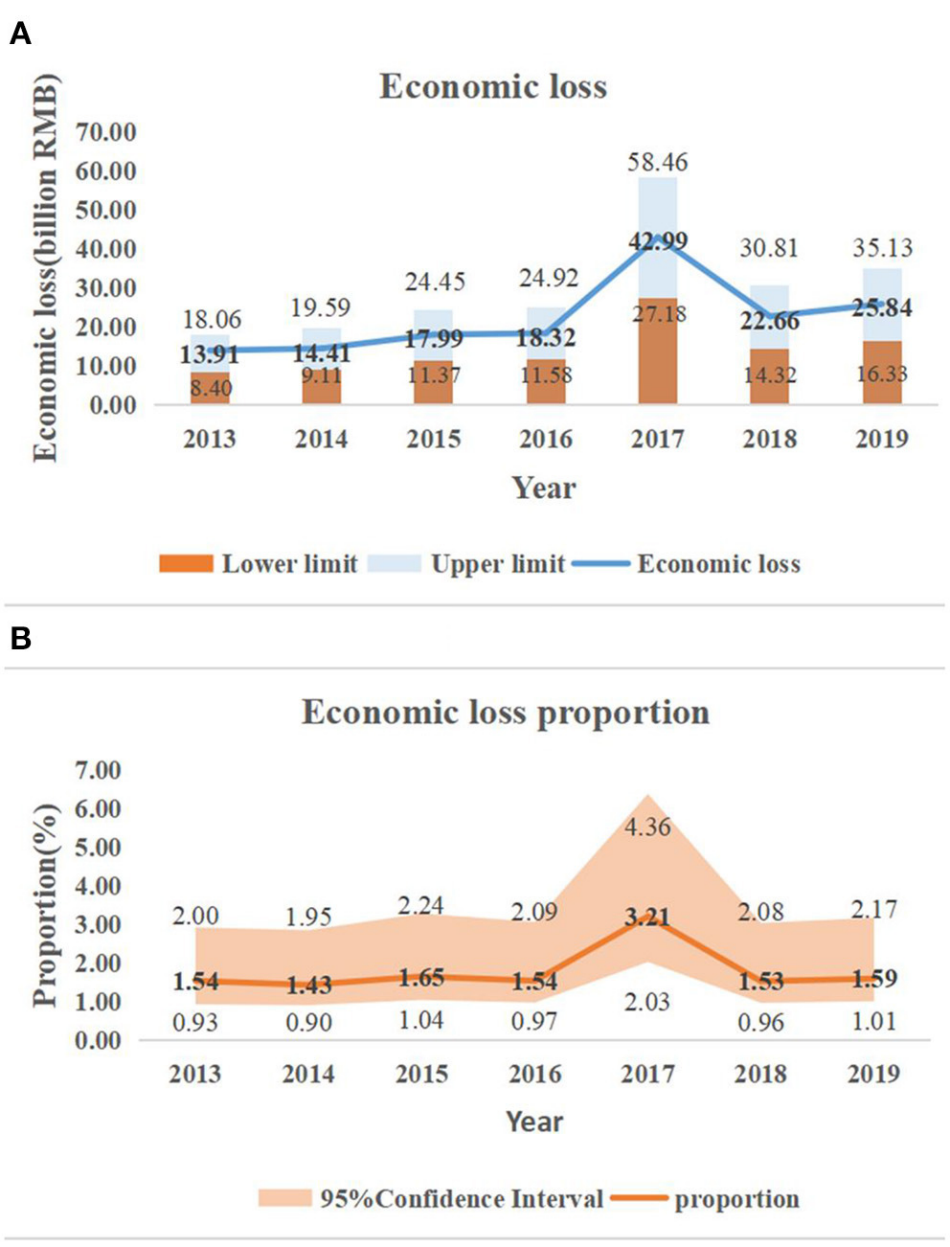
Economic loss from high temperature and the corresponding 95% confidence interval in Wuhan, 2013–2019: **(A)** economic loss; **(B)** proportion of GDP.

The corresponding values of the statistical life in Wuhan over the seven-year period were calculated using Equation (5), as presented in the third column of Table 4. The VSL values related to high temperature varied from 1.57 million RMB in 2013 to 2.24 million RMB in 2019, revealing a generally increasing trend. The progressive increase in VSL indicates the annual improvement in the per capita disposable income level of Wuhan residents. Meanwhile, it also shows the growing willingness-to-pay of residents of Wuhan city to reduce the risk of heat-related deaths. Based on Equation (6), the annual VSL is combined with the corresponding number of premature deaths attributed to high temperature. The outcomes of economic loss from high temperatures and the corresponding proportion in GDP are displayed in Figure 5. The total economic losses between 2013 and 2019 amounted to 156.1 billion RMB (95% CI: 92.28–211.40 billion RMB), accounting for approximately 1.81% (95% CI: 1.14–2.45%) of the GDP of Wuhan in the 7-year period, which turns out to be 8,461.98 billion RMB.

**Table 4:** Value of statistical life in Wuhan between 2013 and 2019.

**Year**	**Per capital disposable income (RMB)**	**VSL (million/person)**	**GDP (billion RMB)**
2013	26,909	1.57	905.13
2014	29,627	1.70	1,006.95
2015	32,478	1.83	1,090.56
2016	35,383	1.96	1,191.26
2017	38,642	2.10	1,341.04
2018	42,133	2.25	1,484.73
2019	46,010	2.42	1,622.32
2013–2019	–	–	8,641.98

Figure 5A displayed that the health economic losses have increased annually between 2013 and 2019, and the economic losses in 2017–2019 have all exceeded the annual average loss (RMB 22.3 billion), contributing to 58.61% of total economic losses. Meanwhile, the largest three values of economic loss were also observed in these 3 years, i.e., 42.99 billion RMB (95% CI: 27.18–58.46 billion RMB), 22.66 billion RMB (95% CI: 14.32–30.81 billion RMB), and 25.84 billion RMB (95% CI: 16.33–35.13 billion RMB). The escalation in health economic losses is associated with a larger frequency and intensity of extremely high temperatures and is further responsible for more premature high temperature-related deaths and a gentle increasing trend in the value of statistical life over time.

Figure 5B reflected the similar trend as Figure 5A. Economic losses were more than 1.5% of the GDP in all year except year 2014. The lower fraction of GDP in 2014 were supposed due to the milder temperatures and less premature deaths from heat exposure as well as the higher increment in GDP in that year compared to other years. The annual average heat economic loss was 1.81% (95% CI: 1.14–2.45%) of GDP from year 2013 to 2019. The Lancet Countdown to China report showed that the economic loss of China from the reduction of labor time due to high temperature was 1.4% of its GDP in 2020 (44), which was 22.7% lower than our estimated economic loss. Thus, Wuhan must develop high-temperature warning measures to reduce its economic losses.

## Discussion

Influence by climate change, high temperatures have been occurring more frequently and with greater intensity. A large number of scholars have developed various models and methods to investigate the health effects of high temperature in Wuhan (31–36). We not only employed meta-analysis to obtain the total impacts but also monetized and quantified the economic burden generated by these mortality impacts. We estimate the economic burden of premature death for selected health endpoints due to exposure to high temperatures to be 156.10 billion RMB (95% CI: 98.28–211.40 billion RMB), accounting for 1.81% (95% CI: 1.14–2.45%) of Wuhan's 2013–2019 GDP, which is higher than the national heat economic losses as a percentage of national GDP published in The Lancet Countdown to China report. To the best of our knowledge, this is the first study to link meta-analysis calculations of relative risk and value of statistical life to quantify high temperature health-related economic losses. It is also an exemplary study in regions seriously threatened by high temperatures such as central China. Other studies of economic losses from high temperatures have adopted method to obtain relative risk directly from urban studies with similar types of conditions to those of the study city or country (45, 46). In this study, epidemiological studies on Wuhan were obtained from four major databases, and relative risks were not employed from epidemiological studies in other cities.

There are some limitations and uncertainties to this study in assessing the economic loss of premature death at high-temperature health endpoints. First, the number of the exposed population selected for the calculation of premature deaths in this study is the registered population of Wuhan, not the permanent population, since Wuhan's all-cause mortality is calculated based on the number of deaths in the registered population (47). Because the registered population is not the only ones exposed to high temperatures, the economic loss from premature death will be underestimated. Second, only five types of causes of death with the most significant effect of high temperature were selected as health endpoints. A study conducted in Seoul, South Korea, indicated a significant correlation between high temperatures and the risk of preterm birth in pregnant women (48). Several studies also illustrated the burden of heat on death from nervous diseases (49), diabetes (50) and mental health (51). Since the relative risks of Wuhan City for the above-mentioned related diseases are not available, a comprehensive assessment of the economic loss from heat exposure is hard to conduct. Once epidemiological data are provided, further studies can be easily performed with more comprehensive and accurate results. Third, we considered only one aspect of the economic impact of heat: premature death. As a result, outpatient costs, medical costs, and lost wages incurred by residents entering emergency care or hospitalization due to sudden illnesses caused by high temperatures are not considered in this paper (5). To ensure the safety of workers under high temperatures, outdoor workers will adopt a work system that reduces the intensity of work or increases the number of breaks, resulting in a reduction in working hours (52); indoor workers will also be less efficient due to the high temperature (52). Average heat-related work productivity loss is about 6.6 days in developing countries in 2016 (53). Seven percent of Australian respondents missed an average of 4.4 days of work during the heat of 2013–2014, and seventy percent were less productive, working for 27.1 h less (12). In such cases, economic loss will also be generated. Therefore, once the hospital admission or emergency data are available, they can be considered in further studies of economic loss of heat.

We believe that the results of such studies serve as a cautionary tale for policymakers. A series of resident mortality risk reduction measures in response to the occurrence of heat events are necessary. An electronic study conducted in Victoria, Australia in 2003 revealed a lack of knowledge among the population regarding thermoregulation, heat risk factors, heat-related illnesses and fan use (54), raising awareness that heat-related knowledge should be strengthened.

It is important to disseminate heat warnings in a timely manner. Issuing heat warning announcements through the media is considered feasible for residents, especially for the elderly population, with radio and television being the two best ways to deliver them (54, 55). Since the elderly and children are more obviously affected by the heat (56), society should give extra attention to these more vulnerable groups (57). In addition, the process of urban construction and development is supposed to accelerate the construction of summer space and cool centers in the city to increase the area for residents to escape from the heat. For example, in 2013, the severest year for high temperatures in Wuhan, the citizens of the city spontaneously went to cooled subway stations to escape the heat, and the subway company stated that they would not refuse such escaping without affecting normal subway operations. In 2019, ~1,400 cool spots in Wuhan city were open to meet the needs of the residents, especially for those of poor households, in accordance with the requirements of civil affairs department of Wuhan. As high temperature momentum is not reducing the trend, Wuhan should consider designating more space throughout the city to escape from the heat.

Different government departments, such as the meteorological department and the health department collaborate to build the emergency response system, complete the simulation implementation and feedback of the system based on big data, and improve the flexibility of the emergency response plan (58). From the perspective of urban construction, ventilation corridors are established according to the prevailing wind direction by relying on the Yangtze River and other lakes, as well as urban roads, parks, and low-density areas in response to the wind brought from the suburbs into the city (59). At the same time, the government accelerated the greening construction focusing on the four banks of the two rivers and the ecological restoration of mountains such as Tortoise Hill and Snake Hill to improve the local microclimate. Since the ecological function protection zones in Wuhan are mainly located in the central part of Huangpi District and the eastern and southwestern parts of Xinzhou District, they have a high resistance valueand these areas can exert ecological benefits, which should be highly valued and protected (60). In addition, greening works in urban parks, roads and scenic areas, as well as forest parks should be renovated to facilitate the mitigation of high temperatures.

## Conclusion

A meta-analysis revealed a relative risk of 1.26 (95% CI: 1.15, 1.39) for the health endpoints. The number of premature deaths due to high-temperature exposures for the five health endpoints in Wuhan was 77,369 (95% CI: 48,906–105,198), and resulted in a loss of 156.10 billion RMB (95% CI: 98.28–211.40 billion RMB), approximately 1.81% (95% CI: 1.14–2.45%) of Wuhan's 2013–2019 GDP. In the context of high global heat events, premature death from heat and the corresponding economic burden deserves close attention. It is suggested that policymakers should also establish appropriate measures to reduce the number of premature heat-related deaths among residents and reduce the economic loss from premature heat-related deaths proportionate to the GDP of the city. Furthermore, this study is a cautionary warning to policy decision-makers for cities and regions facing similar heat threats as Wuhan in the future and is expected to provide valuable management advice in heat-related policymaking.

## Data Availability Statement

The original contributions presented in the study are included in the article/supplementary material, further inquiries can be directed to the corresponding author.

## Author Contributions

SC and JZ: conceptualization, methodology, and writing—original draft preparation. JZ: software, formal analysis, resources, and data curation. JZ and SK: investigation. S-BL and SK: writing—review and editing. S-BL: visualization. SK: supervision. SC: project administration and validation. SC and SK: funding acquisition. All authors contributed to the article and approved the submitted version.

## Funding

This study was supported by the Humanities and Social Science Research Program funded by the Ministry of Education of China (21C10512050). SK research was partially supported by Science Research Program through the National Research Foundation of Korea (NRF) funded by the Ministry of Education (NRF-2021R1A2C1005271).

## Conflict of Interest

The authors declare that the research was conducted in the absence of any commercial or financial relationships that could be construed as a potential conflict of interest.

## Publisher's Note

All claims expressed in this article are solely those of the authors and do not necessarily represent those of their affiliated organizations, or those of the publisher, the editors and the reviewers. Any product that may be evaluated in this article, or claim that may be made by its manufacturer, is not guaranteed or endorsed by the publisher.
